# Cyclic and Sleep-Like Spontaneous Alternations of Brain State Under Urethane Anaesthesia

**DOI:** 10.1371/journal.pone.0002004

**Published:** 2008-04-16

**Authors:** Elizabeth A. Clement, Alby Richard, Megan Thwaites, Jonathan Ailon, Steven Peters, Clayton T. Dickson

**Affiliations:** 1 Centre for Neuroscience, University of Alberta, Edmonton, Alberta, Canada; 2 Department of Psychology, University of Alberta, Edmonton, Alberta, Canada; 3 Department of Physiology, University of Alberta, Edmonton, Alberta, Canada; University of Southern California, United States of America

## Abstract

**Background:**

Although the induction of behavioural unconsciousness during sleep and general anaesthesia has been shown to involve overlapping brain mechanisms, sleep involves cyclic fluctuations between different brain states known as active (paradoxical or rapid eye movement: REM) and quiet (slow-wave or non-REM: nREM) stages whereas commonly used general anaesthetics induce a unitary slow-wave brain state.

**Methodology/Principal Findings:**

Long-duration, multi-site forebrain field recordings were performed in urethane-anaesthetized rats. A spontaneous and rhythmic alternation of brain state between activated and deactivated electroencephalographic (EEG) patterns was observed. Individual states and their transitions resembled the REM/nREM cycle of natural sleep in their EEG components, evolution, and time frame (∼11 minute period). Other physiological variables such as muscular tone, respiration rate, and cardiac frequency also covaried with forebrain state in a manner identical to sleep. The brain mechanisms of state alternations under urethane also closely overlapped those of natural sleep in their sensitivity to cholinergic pharmacological agents and dependence upon activity in the basal forebrain nuclei that are the major source of forebrain acetylcholine. Lastly, stimulation of brainstem regions thought to pace state alternations in sleep transiently disrupted state alternations under urethane.

**Conclusions/Significance:**

Our results suggest that urethane promotes a condition of behavioural unconsciousness that closely mimics the full spectrum of natural sleep. The use of urethane anaesthesia as a model system will facilitate mechanistic studies into sleep-like brain states and their alternations. In addition, it could also be exploited as a tool for the discovery of new molecular targets that are designed to promote sleep without compromising state alternations.

## Introduction

Sleep is a condition of altered consciousness in which there is a reduction of sensory awareness, behavioural output and metabolic activity. Sleep is expressed as a circadian rhythm under neural control and appears to be essential for survival [1]. Although sleep takes up a significant portion of our biological existence, its functional purpose (both ecological and physiological) remains unclear. One of the fundamental mysteries of sleep is the production of brain state alternations, i.e., the REM/nREM cycle.

Sleep in mammals and birds involves two main stages as measured by field potential (EEG) recordings: 1) quiet, slow-wave, or nREM sleep, which is characterized by large-amplitude and slow cortical rhythms; and 2) active or REM sleep, which is characterized by low-amplitude and faster cortical rhythms. The latter stage is also known as paradoxical sleep since the EEG patterns are similar to those present during the alert awake state. Alternations between nREM and REM sleep occur at regular intervals throughout a continuous sleep episode. The functional relevance of these stages and their alternations is unknown, although depriving subjects of either stage can induce detrimental effects independent of sleep loss itself, and a rebound amount of time spent in the deprived state in subsequent sleep episodes [1]–[5].

Although progress towards an understanding of 1) the mechanisms, and 2) the functional relevance of state dependent patterns of brain activity and their alternations in sleep has certainly been made since their discovery in 1953 [6], research in the field has been made difficult by the lack of a model of sleep state alternations other than sleep itself. For certain experimental paradigms the naturally sleeping animal presents both technical and ethical obstacles. This has undoubtedly limited our scientific progress in this field. The most common model for sleep has been anaesthesia, which produces a behavioural condition not unlike natural sleep [7]. In fact, “sleep” is the most common metaphor or analogy used for anaesthesia by physicians and laypersons alike [8]. The similarities between sleep and anaesthesia include: a subjective loss of consciousness, reduced sensory awareness, and a reduction in behavioural responsiveness. Moreover, recent research has provided evidence that there is a considerable overlap in the physiological mechanisms of anaesthesia and the induction of the sleeping state (reviewed in [9]). Of course, differences between the two conditions are obvious, including the dependence of anaesthesia on circulating levels of pharmacological agents, the inability to rouse anaesthetized subjects and, perhaps most importantly, the lack of cyclical variability of brain states while under the influence of most anaesthetics

Here we report that rats anaesthetized with urethane demonstrate spontaneous and cyclical alternations of brain state that resemble sleep state alternations. We systematically evaluated their similarity by comparing their EEG components, evolution, time frame, physiological correlates, pharmacology and dependence upon ascending neuromodulatory brain systems. Our results suggest that the pharmacological action of urethane in the brain closely mimics the physiological mechanism(s) for the maintenance of the natural sleeping state in rats and thus implicates this preparation as a valid model for the investigation of brain mechanisms giving rise to cyclical state alternations.

## Results

### Spontaneous and rhythmic brain state alternations under urethane anaesthesia

**Figure 1:**
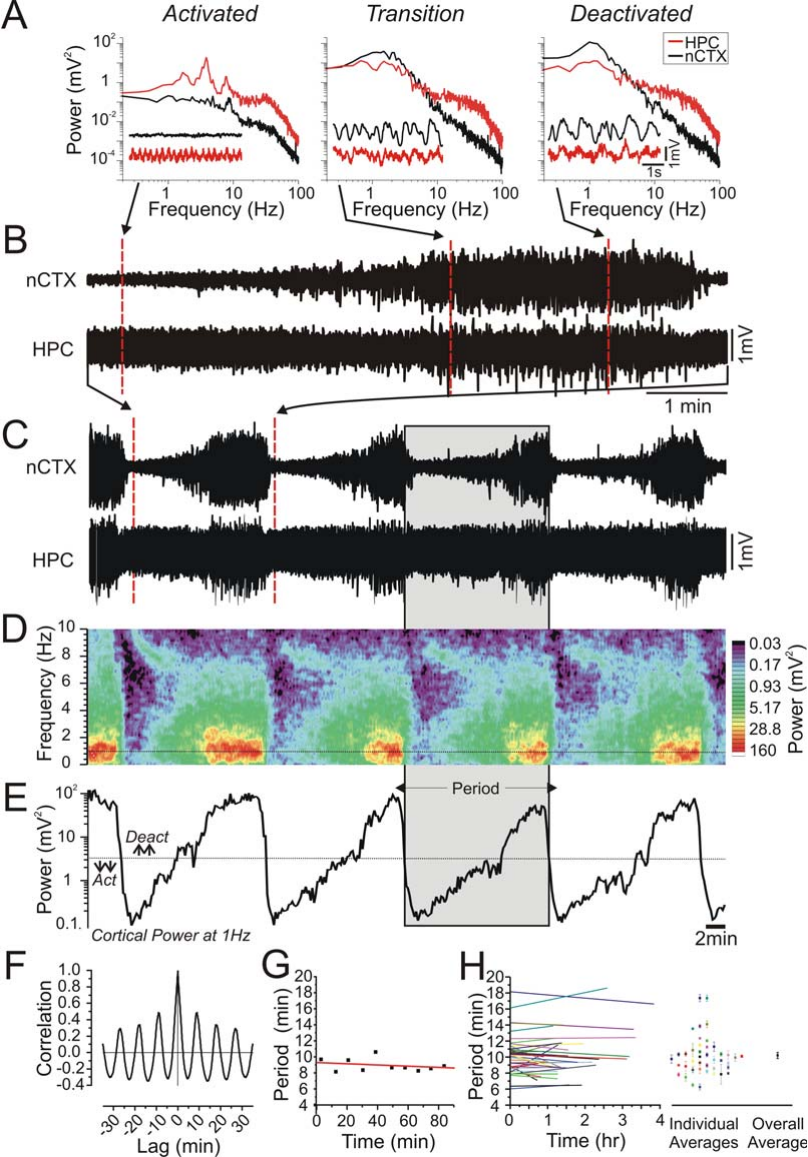
Urethane anaesthetized animals displayed spontaneous and cyclic alternations of brain state. A) Spectra and raw EEG traces of different spontaneous states under urethane. During the activated state, the nCTX showed low voltage fast activity and the HPC showed prominent theta at ∼4 Hz. During the transition state, there was an increase in overall power with a shift to lower frequencies at both sites; raw EEG traces showed irregular and moderately high amplitude activity. During the deactivated state, there was an even more prominent shift to low (∼1 Hz) frequencies with a further increase in overall power and raw EEG traces displaying prominent rhythmic high amplitude slow activity. B) Continuous EEG traces of a spontaneous transition from activated to deactivated patterns. Positions from where the expanded traces in A were taken are shown. The transition between activated and deactivated patterns was marked by a gradual increase in the amplitude of the signals whereas the transition between deactivated and activated patterns was more abrupt. C), Continuous EEG traces over an even longer time scale demonstrated a regular and cyclic alternation of state as observed by the fluctuations in amplitude. The position from where traces in B were taken are shown. D) A spectrographic representation of the neocortical trace shown in C. The most prominent fluctuation of power was centered at a frequency of circa 1 Hz. E) Plot of power at 1 Hz from the spectrograph in D. The power values showed a cyclic fluctuation in amplitude continuing over the entire time of recording with an average period of ∼9 minutes. F) Autocorrelation of power values from the experiment shown in (E) that showed a prominent rhythmic fluctuation at a similar period (9 min). G) Scatter plot of alternation period across time. The period length for the experiment illustrated remained stable as shown by the linear fit with a slope value not significantly different from zero (p = 0.46). H) The left panel shows regression lines for cycle periods across time for all experiments having 6 or more cycles. Different experiments are represented by different colored lines and demonstrate a general lack of variation within, but some variation across, animals. Thirty eight of the forty one animals displayed here showed no significant regression at a p level of 0.05. The right panel is a scatter plot of the average periods for each experiment (equivalent colors to the right panel) and the overall average (10.3±0.4 min) across all experiments.

In long term (>45 minute) field (EEG) recordings made from neocortical and hippocampal sites in urethane-anaesthetized rats, a spontaneous and highly regular alternation of electrographic state between activated and deactivated patterns was noted. Activated EEG patterns consisted of low amplitude fast activity at neocortical sites concomitant with theta (rhythmic 3–5 Hz) activity at hippocampal sites while deactivated EEG patterns were characterized by large-amplitude slow oscillatory (∼1 Hz) activity at both neocortical and hippocampal sites (Figure 1A). As previously reported [10], the spontaneous evolution between these states was stereotyped and involved a gradual transition from the activated to the deactivated state (5.23±0.60 minutes, range: 2.75 to 8.58, n = 10) and then a rapid (52±4 s, range: 34 to 78, n = 10) shift back into the activated state (Figure 1B). This cycle repeated itself in a stable and consistent rhythmic fashion as demonstrated by observation of the amplitude fluctuations of long-duration plots of raw EEG traces (Figure 1C) and spectrographic plots computed on the same EEG traces (Figure 1D). Within spectrograms, the highest power fluctuations were centered at a frequency of 1 Hz (especially in the neocortex) so the period and rhythmicity of the alternations were systematically characterized by extracting this frequency band across time (Figure 1E). The precise time points of alternations between states were denoted by calculating a threshold value (corresponding to the saddle of the bimodal distribution of the power at 1 Hz across time) that separated activated from deactivated patterns (values below the threshold were considered to be activated while those above were considered to be deactivated). Autocorrelations of the de-trended power at 1 Hz were highly rhythmic (Figure 1F) and successive periods maintained a consistent duration across time (Figure 1G). This consistency was observed in the vast majority of experiments (38 of 41 see Fig 1H) by showing that least-squares linear fitting of the period durations across time did not result in significant slope values (p>0.05). All of these results were consistent with a systematic and stable cyclic process.

These rhythmic state alternations were a consistent finding in 79 out of 83 animals and had an average period of 10.5±0.3 minutes. Of the four animals that did not show this effect, one showed a consistent deactivated electrographic pattern and the other three showed irregular (arrhythmic) and inconsistent alternations between activated and deactivated patterns.

### State alternations under urethane anaesthesia were not a result of fluctuating anaesthetic levels

Although previous investigators have noted state changes under urethane anaesthesia, none have systematically described the consistent, spontaneous, and rhythmic alternations reported here (*cf.* [11] p. 36). Indeed, state changes under urethane have typically been ascribed to variations in anaesthetic level [12]–[14] even though prior studies of the metabolism of urethane have shown a constant and non-fluctuating rate of decline over time as measured in the blood [15], [16]. In order to systematically assess the idea that state alternations were a result of variations in anaesthetic level we 1) examined the effects of supplemental doses of urethane on state alternations and 2) monitored anaesthetic level using a behavioural assay across state alternations.

**Figure 2:**
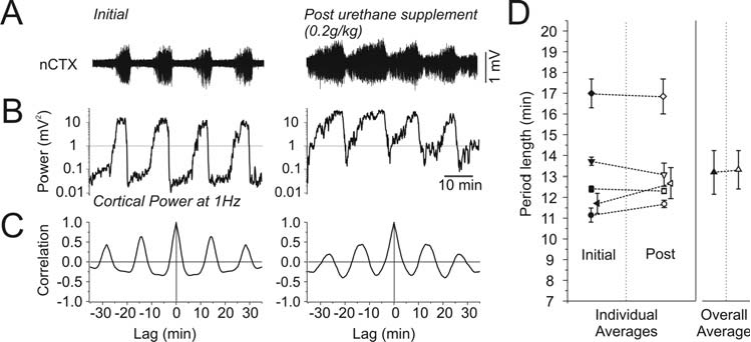
The rhythmicity and periodicity of state alternations under urethane were not affected by moderate increases in anaesthetic dosage. A) Long duration raw cortical EEG traces demonstrating the electrographic effects of a moderate increase in the depth of urethane anaesthesia. Although the overall amplitude of the signal increased following the supplemental dose, state alternations (apparent as rhythmic changes in signal amplitude) were still observed. B) Spectrographic power at 1 Hz for the traces shown in A. Following the supplemental dose, power continued to fluctuate at a similar periodicity although there was an increase in the overall power in addition to the time spent in the deactivated state (and consequently a decrease in the time spent in the activated state). C) Autocorrelation of power values in B demonstrating similar rhythmicity before and after supplemental urethane administration. D) Individual (paired for animal across conditions by symbol and line) and overall averages demonstrating the consistency of alternation period duration before and after the supplemental doses of urethane across all experiments. Neither the individual nor the overall averages were significantly different.

Supplemental doses of urethane (ranging from 0.07 to 0.27 mg/kg), while decreasing the relative amount of time spent in the activated state (8.9±2% for every 0.1 mg/kg administered, n = 5), did not alter either the presence or the rhythmicity of state alternations *per se* (Figure 2). No significant differences were observed for the duration of alternation periods pre- and post-supplement in any individual experiment (p≥0.15) nor overall (p = 0.68). This suggests that the cyclicity of state alternations is not affected by the absolute concentration of urethane in the blood.

**Figure 3:**
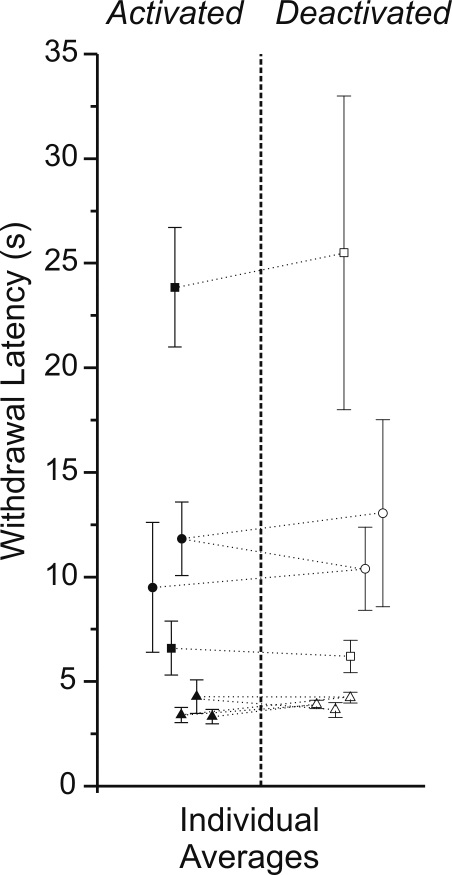
Anaesthetic level was comparable across state alternations. A) Individual (paired for animals across states by symbol) and overall averages of withdrawal latency across activated and deactivated states in response to a ramped infrared beam applied to the tail or hindpaw. Withdrawal latencies were comparable across states and neither the individual nor overall averages were significantly different.

We also found that a behavioural assay of anaesthetic level was consistent across states. In initial experiments, we assessed withdrawal to sharp and consistent pressure applied on the hind paw pad within each state. Although we could find no differences using this technique we further (and more systematically) examined level of anaesthesia by directly measuring latency to withdrawal using a ramped heat tail-flick apparatus (see Methods section). Although the intensity of the infrared beam had to be maximized in order to elicit even a weak but measurable withdrawal response (consistent with a surgical plane of anaesthesia) a pair-wise t-test performed on temporally adjacent stimulation sets across all animals revealed no significant differences in withdrawal latency between activated and deactivated states (Figure 3, n = 10, p = 0.31). Thus, state alternations were not due to variations in anaesthetic level.

### EEG states and their alternations under urethane anaesthesia were comparable to those of natural sleep

A novel interpretation of these rhythmic and regular fluctuations in brain state under urethane is that they reflect overlapping mechanisms to those expressed during natural sleep. Indeed, prior measurements of the duration of REM/nREM sleep cycles in rats have a distribution with a mean and modal value between 9.5 and 13.5 minutes [17]. This timing overlaps extremely well with the distribution that we found for state alternations under urethane.

**Figure 4:**
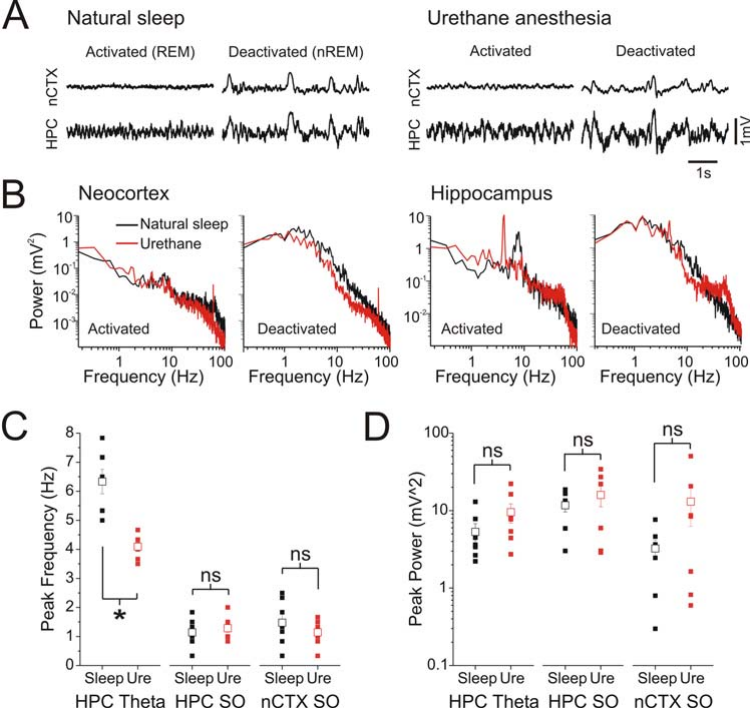
Raw and spectral characteristics of forebrain EEG during activated and deactivated states were similar across during natural sleep and urethane anaesthesia. A) Expanded neocortical and hippocampal EEG traces across natural sleep and urethane anaesthesia in the same animal showing examples of activated and deactivated patterns in both situations. Regardless of condition, activated and deactivated patterns were highly similar. B) Spectra of cortical and hippocampal EEG traces overlaid across conditions for the same electrographic patterns. Although the peak frequency of hippocampal theta power during REM was at a higher frequency (∼7 Hz) than during the activated state under urethane anaesthesia (∼4 Hz), all other spectra across conditions appear highly similar. Scatter plot of C) peak frequencies and D) power (right panel) for neocortical and hippocampal signals during the activated and deactivated state for each animal. Except for the peak frequency of hippocampal signals during activated patterns, there were no significant differences across natural sleep and urethane anaesthesia.

To directly evaluate the similarity of the two conditions, we performed recordings in naturally sleeping rats that were later anaesthetized with urethane (Figure 4). The raw electrographic characteristics of REM sleep were highly similar to those demonstrated in the activated state under urethane anaesthesia, with the cortex displaying low-voltage fast activity (LVFA) and the hippocampus eliciting a prominent theta rhythm. The spectral characteristics of both states, assessed by comparison of peak frequencies and power, were likewise similar although the peak frequency of theta was significantly higher on average in REM (6.4±1.2 Hz) than in the activated state during urethane (4.1±0.1 Hz, p = 0.001) (Figure 4A, B, C). Likewise, during slow-wave sleep and the deactivated state under urethane anaesthesia, both cortical and hippocampal traces were dominated by the slow oscillation [10] and spectra showed overlapping distributions without significant differences in either peak frequency or power across these conditions (Figure 4A, B, C).

**Figure 5:**
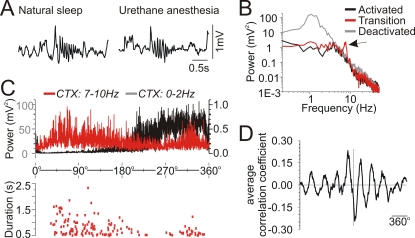
Transition phase between activated and deactivated patterns was characterized by cortical spindling across both natural sleep and urethane anaesthesia. A) An expanded example of a cortical spindle oscillation recorded during natural sleep (left) and a similar pattern recorded at the same electrode during the transition phase under urethane anaesthesia (right). B) Superimposed spectral plots of cortical EEG taken during activated, transition and deactivated states in a urethane-anaesthetized animal. Note the spectral peak in the transition spectra centered at ∼8 Hz (spindle frequency). C) Superimposed spectrographic power at low (0 to 2 Hz: black) and spindle (7 to 10 Hz: red) frequencies (top panel), and the simultaneous occurrence and duration of spindles across the evolution of all state alternations for one full experiment (bottom panel). Note the augmented presence of spindling activity at the transition points between activated and deactivated patterns. D) Average (across all experiments) of the cross correlation function between 1 Hz and spindle (7–9 Hz) spectrographic power (n = 5; standardized cycle in degrees). The temporal relationship of spindling to slow oscillatory activity at 1 Hz patterns showed a consistent lag of approximately half a period length (∼180 degrees).

In addition, another electrographic similarity was apparent during the transition period between activated and deactivated patterns across both sleep and urethane anaesthesia. During this period, the cortical EEG traces showed spindle activity, often with a characteristic K-complex-like spike followed by an envelope of high frequency (7–15 Hz) oscillations lasting longer than 0.5 s. As shown in Figure 5A, these events were remarkably similar across both natural sleep and urethane anaesthesia as recorded in the same animals. In fact, during urethane anaesthesia, spindle frequencies were a consistent facet of the transition period as demonstrated by a spectral peak in the 7–10 Hz range (Figure 5B). In long-duration recordings under urethane, spindle events were more probable and of higher duration in the intervening period between activated and deactivated states (Figure 5C). As well, increases in spindle frequency power (bandwidth 7–10 Hz, average: 8.2±0.4 Hz, n = 5) consistently led increases in 1 Hz (i.e. slow oscillation) power at an average lag of 170 degrees (i.e. at approximately the half-cycle point) with respect to the rhythmic activated-deactivated state alternation (Figure 5C,D).

**Figure 6:**
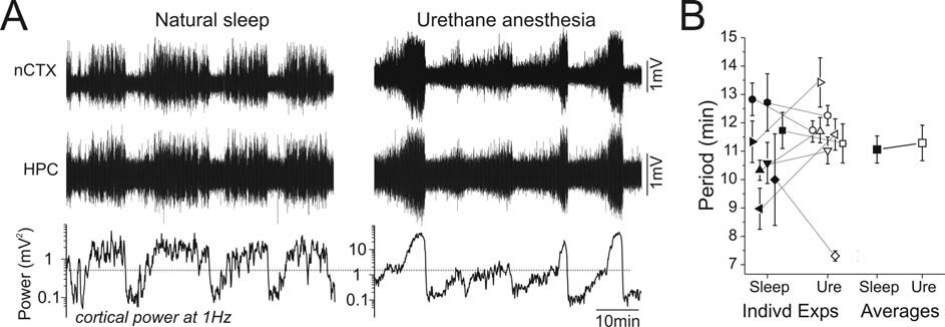
The timing of cyclic EEG state alternations was highly similar across naturally sleeping and urethane-anaesthetized conditions. A) Long-duration EEG traces during a continuous natural sleep episode (left panel) and subsequently from the same animal under urethane anaesthesia (right panel). Regular fluctuations between REM and nREM sleep stages were highly comparable in their timing to the state alternations later observed under urethane. Plotted on the same time scale underneath the raw traces is the respective spectrographic power at 1 Hz extracted from the cortical signals. These fluctuations demonstrated a similar rhythmicity across both conditions. B) Scatterplot representing period lengths of alternations for all animals under both naturally sleeping and urethane anaesthetized conditions. There was a remarkable similarity in the distribution and average length of alternations for each animal as well as for the overall average for natural sleeping and urethane anaesthetized conditions.

Not only were the electrographic aspects of individual states similar across natural sleep and urethane anaesthesia, but the average alternation period between these states was also highly comparable (Figure 6). The same animals recorded across conditions showed an average cycle length of 11.1±0.5 minutes in natural sleep and 11.3±0.6 minutes when later recorded under urethane anaesthesia (Figure 5B). This difference was not significant (n = 8, two-tailed pair-wise t-test, p = 0.73). Perhaps not surprisingly, however, there was significantly greater variance in cycle length during sleep as compared to urethane anaesthesia (19.3±2.3 s in natural sleep versus 7.2±0.6 s in urethane anaesthesia, n = 8, pair-wise t-test, p = 0.001). This could have reflected a stereotypy of alternations mechanisms under urethane or disturbances of sleep cycling by arousal into wakefulness, or both.

### State alternations under urethane anaesthesia showed similar physiological correlates to those of natural sleep

Our EEG data suggested a similarity in the brain mechanisms responsible for both the induction and maintenance of unconsciousness across both sleep and urethane anaesthesia. To assess the similarities of other physiological processes know to co-vary with sleep states we evaluated eye movements via electrooculography (EOG), muscle tone via electromyography (EMG), heart rate via electrocardiography (EKG) and respiration rate [6], [18]–[21] during EEG state alternations under urethane anaesthesia.

EOG signals were non-existent under urethane anaesthesia and showed no differential activity across states (n = 3). Indeed, in contrast to sleep, rats under urethane typically maintained open eyelids.

**Figure 7:**
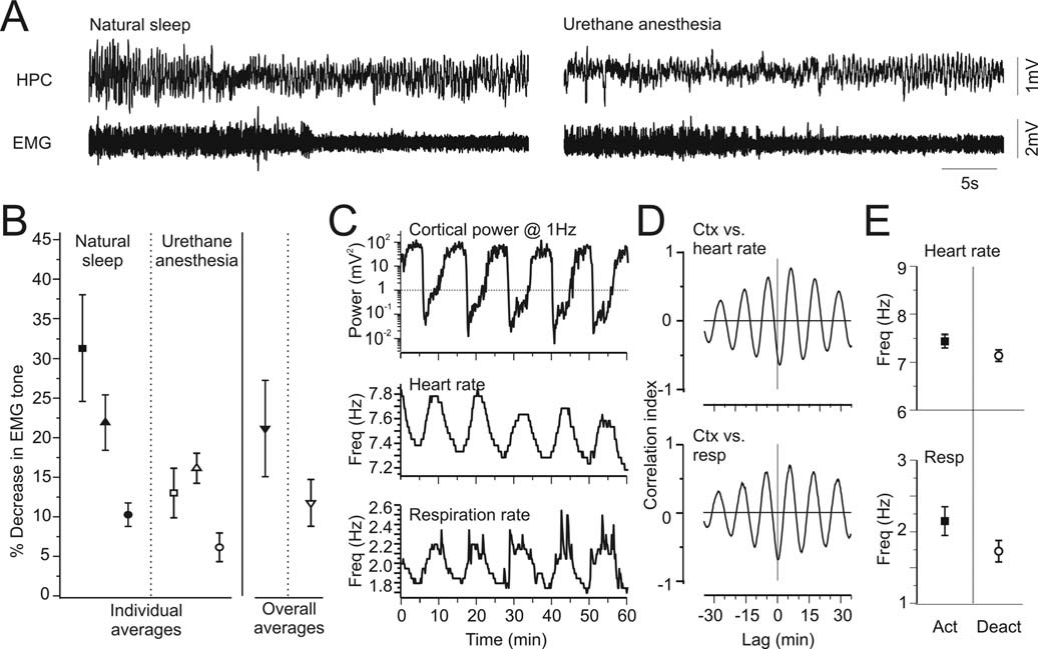
Physiological correlates of EEG state changes were similar across naturally sleeping and urethane-anaesthetized conditions. A) Simultaneous recordings of hippocampal field activity and neck EMG in naturally sleeping (left panel) and urethane anaesthetized (right panel) conditions in the same animal. Declines in EMG tone with transitions from nREM to REM sleep were also apparent under urethane anaesthesia with transitions from deactivated to activated EEG patterns. B) The average percentage loss of EMG amplitude across these transitions was consistent and significantly different across both natural sleep and urethane anaesthesia in the same animals. C) Simultaneous extraction of cortical spectrographic power at 1 Hz (top panel), heart rate (middle panel) and respiration rate (lower panel) demonstrating concomitant fluctuations. Increases in both heart and respiration rates appeared during the lowest EEG power readings (i.e. the activated state). As well, during the activated state, the peak frequency of respiratory cycle tended to show greater variation. D) Fluctuations in heart (top) and respiration rates (bottom) were rhythmically correlated with state changes as shown in the cross correlation of these variables to cortical power at 1 Hz. E) Summary data across experiments showing significant increases in both heart (top) and respiration rates (bottom) when comparing the activated to the deactivated state.

Our EMG recordings during urethane, however, did show changes consistent with natural sleep. In 3 of the 5 animals that had functional EMG recordings during natural sleep, we observed similar and significant (all pair-wise comparisons significant at p<0.01) decreases in EMG tone during transitions from deactivated to activated states during subsequent recordings during urethane (Figure 7A, B). The average decline across deactivated to activated states was 21.2±6.1%, during natural sleep as compared to 11.8±3.0% under urethane. This was despite the fact that urethane is known to induce muscular atonia itself [22], which was supported in our experiments by significantly lower average peak-to-peak values of EMG during urethane (1.36±0.55 mV) as compared to sleep (2.67±1.06 mV: one-tailed pair-wise t-test, p = 0.04, n = 5),, Consistent with a depression of EMG tone generally, the decline under urethane was smaller than that in natural sleep although this difference was not statistically significant (p = 0.09). In the remaining two animals, there was no change in the EMG tone under urethane across states but this failure may have reflected a floor effect due to a general depression of muscular tone. None of our animals demonstrated phasic events characterized by large muscular twitches during the activated state.

Lastly, and again similar to natural sleep, consistent and significant increases in both heart (0.29±0.06 Hz: 4.1±0.8%) and respiration (0.42±0.08 Hz: 24.1±4.1%) rates were concomitant with transitions from deactivated to activated states under urethane anaesthesia (Figure 7C,D,E). Furthermore, in 3 of 4 animals, respiration rate became significantly more irregular as characterized by higher variability (more frequent frequency shifts) during activated states (chi^2^ analysis, p<0.05, n = 3 and p = 0.90, n = 1). Thus, alternations in brain state exhibited under urethane anaesthesia were correlated with physiological changes consistent with those exhibited during natural sleep.

### State alternations under urethane anaesthesia depended on brain systems known to influence natural sleep state alternations

State alternations during sleep are known to be due to forebrain fluctuations in the release of endogenous acetylcholine (ACh) emanating from differential activity in ascending brainstem arousal systems (reviewed in [20], [23]–[26]). We sought to test the cholinergic dependence of state alternations under urethane by: 1) demonstrating and contrasting the pharmacological effects of interfering with central cholinergic versus monoaminergic neurotransmission, 2) demonstrating the effects of temporary inactivation of the major cholinergic afferent of the neocortex and hippocampus, the basal forebrain region, and 3) demonstrating the effects of electrical stimulation of ascending cholinergic brainstem nuclei such as the pedunculo pontine tegmental nucleus (PPT) and lateral dorsal tegmental nucleus (LDT) that are proposed to be elements of the pacemaking circuit for brain state alternations in sleep [27], [28].

**Figure 8:**
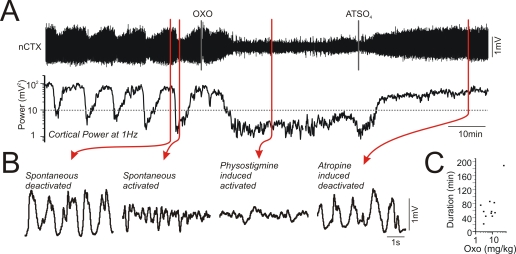
State alternations were dependent upon central muscarinic neurotransmission. A) Long duration cortical EEG traces in addition to spectrographic cortical power at 1 Hz demonstrating the effects of agonism and subsequent antagonism of muscarinic receptor mediated transmission. Following an i.p. injection of oxotremorine (4.0 mg/kg) spontaneous alternations between activated and deactivated states were abolished in favor of the activated state. This induced activated state was abolished in favor of the deactivated state with a subsequent i.p. injection of atropine sulfate (ATSO_4_: 50 mg/kg). B) Expansions of EEG traces from neocortical sites show the similarity of activated and deactivated patterns induced by cholinergic agonism and antagonism, respectively. C) A scatter plots demonstrating the duration of the oxotremorine effect as a function of dosage. The effects of oxotremorine were longer than those of physostigmine (Figure 6C). As in Figure 6, the effects of atropine showed no reversal.

Systemic manipulations using the acetylcholinesterase inhibitor physostigmine (2.39±0.9 mg/kg, n = 16) promoted a long lasting activated forebrain state (36.3±6.2 minutes), similar to the spontaneous activated state (Figure S1). Treatments using the muscarinic receptor agonist oxotremorine (11.17±3.96 mg/kg, n = 11) also elicited activated forebrain EEG patterns similar to spontaneous activated patterns (Figure 8A, B) and this effect had a longer duration (70.1±13.2 minutes, Figure 8A, C). Treatments with the muscarinic antagonist atropine sulphate (50 mg/kg, n = 17) produced a deactivated state similar in spectral characteristics to spontaneous deactivated patterns (Figure 8B and Figure S1). This effect appeared non-reversible since no washout occurred over recording times as long as 100 minutes (average recording duration: 46.9±6.9 minutes). All of the above manipulations resulted in the abolition of state alternations, although as physostigmine was metabolized, a return to alternations into the deactivated state began taking place (Figure S1). These results demonstrate that spontaneous alternations of state in the urethane-anaesthetized animal, like natural sleep, are also dependent on central muscarinic mechanisms.

**Figure 9:**
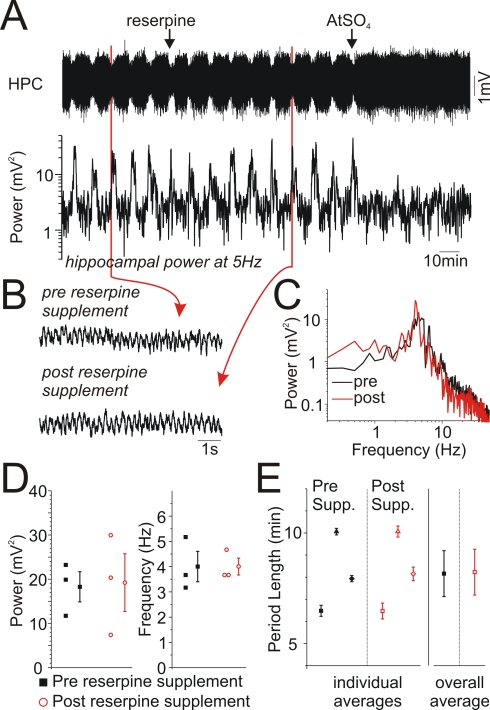
Monoaminergic depletion affected neither the activated state nor the alternations between states under urethane anaesthesia. A) Continuous hippocampal EEG traces and spectrographic power at theta frequencies under urethane anaesthesia following reserpine (5 mg/kg) pretreatment. Alternations between states were obvious as fluctuations in the amplitude of the raw EEG in addition to the spectrographic theta power (5 Hz). A further supplemental i.p. administration of reserpine (5 mg/kg) was without effect upon either the activated state or the alternations between states. However, a subsequent i.p. injection of atropine (ATSO_4_: 50 mg/kg) completely abolished the activated state and subsequent alternations between state. Hippocampal B) EEG expansions and C) spectra from before and after reserpine supplement demonstrating intact theta activity in both cases. D) Scatterplots of peak power and frequencies for activated states pre and post reserpine supplement. There were no significant differences between pre and post supplement groups. E) Scatterplots of period lengths pre and post reserpine supplements demonstrating that alternations and rhythmicity were not affected by reserpine.

A major difference between forebrain activation during wakefulness and during REM sleep is the role of ascending monoaminergic systems, most notably the noradrenergic system [29], [30]. In order to eliminate any possible contribution of these non-cholinergic systems in the elicitation of activated states and state alternations under urethane, we depleted monoaminergic vesicular stores by pre-treating animals with reserpine (5 mg/kg) 14–18 hours prior to our experiments. The pharmacological action of reserpine was confirmed by behavioural observations in the intervening period for sedation, ptosis and akinesia [31]–[33]. Despite successful pre-treatments with reserpine, all subsequently anaesthetized animals displayed spontaneous and rhythmic alternations of brain state demonstrating both activated and deactivated patterns (Figure 9). Furthermore, a supplemental dose of reserpine (5 mg/kg) during anaesthesia affected neither the occurrence nor characteristics of the activated state nor did it affect the ongoing state alternations. Measurements were limited to hippocampal traces as variations in theta frequency and power are a sensitive index of the level of forebrain activation [34]. As shown (Figure 9B, C, D), raw traces and spectral components showed no differences pre- and post-reserpine supplement (p≥0.926 for both power and frequency measures). As well, the period length prior to supplement (8.16±1.04 minutes) was not significantly different from that measured post-supplement (8.23±1.04 minutes: p = 0.44, n = 3) (Figure 9E). Therefore, individual states and their alternations under urethane anaesthesia were not dependent upon the integrity of monoaminergic transmission as they are during the waking state.

**Figure 10:**
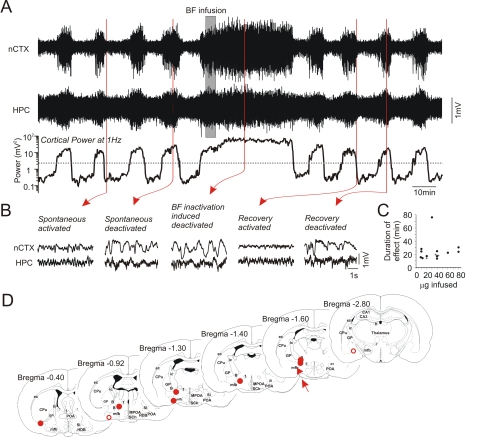
Local pharmacological inactivation of the basal forebrain region reversibly induces a deactivated state and temporarily abolishes state alternations. A) Continuous long duration cortical and hippocampal EEG traces together with spectrographic cortical power at 1 Hz during an infusion of lidocaine in the basal forebrain (BF). Spontaneous state alternations were temporarily abolished in favor of a continuous activated state following inactivation of the BF. B) Expanded EEG traces from neocortical and hippocampal sites prior and following the infusion. Deactivated patterns were elicited during inactivation of the BF and these effects washed out with time. C) Duration of evoked deactivated activity as a function of the amount of lidocaine infused in the BF. D). Histological representation of infusion sites. Open circles indicate ineffective sites. Site marked with arrow and triangle denotes experiment shown in A and B. Abbreviations: B: basal nucleus, CPu: caudate putamen, ec: external capsule, f: fornix, GP: globus pallidus, HDB: horizontal limb of the diagonal band of Broca, ic: internal capsule, mfb: medial forebrain bundle, MPOA: medial preoptic area, POA: preoptic area, SCh: suprachiasmatic nucleus, SI: substantia innominata.

Since central cholinergic mechanisms appeared to be a major factor in controlling state alternations we next sought to investigate the role of structures in the brain that influence forebrain levels of acetylcholine. The direct source of ACh to the neocortex and the hippocampus emanates from nuclei in the BF that have also been implicated in the elicitation of activated patterns of the EEG during REM sleep [29]. We assessed the role of these nuclei by temporarily inactivating these sites using direct intracerebral infusions of 1 – 4% solutions of lidocaine (22.5±4.5 µg: n = 8). Infusions centered at sites such as the magnocellular preoptic nucleus, the substantia innominata, the horizontal limb of the diagonal band, the basal nucleus, and the ventral aspect of the globus pallidus (for a representation of the actual histological sites see Figure 10D) produced a rapid (61±10 s) onset of a continuous deactivated pattern in the EEG of both the nCTX and HPC that lasted for an average duration of 26.1±7.4 min and resembled patterns elicited during spontaneous deactivation (Figure 10A, C). During this time, no alternations of state took place. Following washout, state alternations returned. Additional equivolume infusions of lidocaine subsequent to washout at sites promoting deactivated states produced identical effects to the initial infusion (n = 2) whereas increased volumes of infusion (n = 5) resulted in an increase of time spent in the deactivated state. In cases in which infusion sites were outside of the BF (placements too lateral or ventral), forebrain EEG was not altered (n = 2, for histological sites see Figure 10D). Thus, as in sleep, the activity of specific nuclei within the BF region appears to be necessary for the expression of activated states and for the alternation of states under urethane anaesthesia.

Although the basal forebrain (BF) is the direct source of forebrain acetylcholine, it is the cholinergic nuclei in the brainstem, such as the PPT and LDT, together with other nearby structures, such as the lateral pontine tegmentum, venterolateral periacquaductal gray, sublaterodorsal nucleus, and precoeruleus, which are thought to constitute the essential pacemaker for sleep state alternations themselves [20], [23], [27], [28], [35]. Therefore, we electrically stimulated these sites in the brainstem in an attempt to disrupt the ongoing state alternations under urethane.

**Figure 11:**
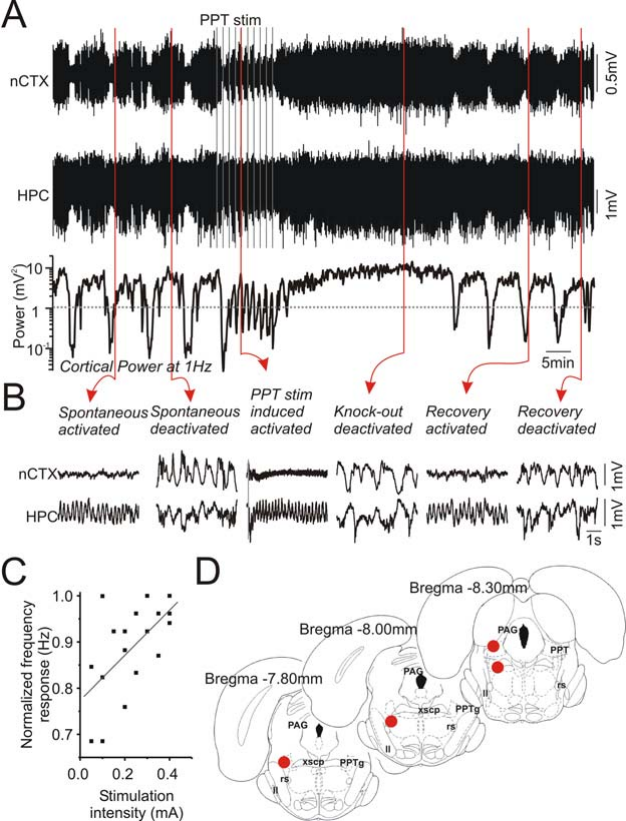
Stimulation trains applied to the pedunculo-pontine tegmentum temporarily abolished alternations of forebrain state. A) Continuous cortical and hippocampal EEG traces and the spectrographic cortical power at 1 Hz demonstrating the effects of stimulation of the pedunculo-pontine tegmental (PPT) region. Subsequent to a series of moderate stimulation trains, which were each effective in promoting an activated forebrain EEG during application, there was a transient suppression of forebrain state alternations. B) Expanded EEG traces from neocortical and hippocampal sites demonstrate that activated patterns were elicited via stimulation and that deactivated patterns follow a stimulation train. C) Scatterplot and linear fit of frequency as a function of the stimulation intensity in the PPT showing a significant (p<0.01) relationship between stimulation intensity and the peak frequency of theta activity recorded in the hippocampus. The frequency was normalized across experiments to the maximal frequency of theta elicited in each. D) Summary of histological findings for the site of stimulation across experiments. Abbreviations: ll: lateral lemniscus, PAG: periaqueductal gray, PPT: pedunculo pontine tegmental nucleus, rs: rubrospinal tract, xscp: decussation of the superior cerebellar peduncle.

As previously reported [36], [37], stimulation trains delivered to sites in or near the PPT resulted in the immediate elicitation of activated patterns in forebrain EEG (Figure 11A, B). As noted by these researchers, an increase in stimulation intensity resulted in a linear increase in the degree of forebrain activation as indexed by the peak frequency of hippocampal theta activity (Figure 11C). More importantly, however, directly following even a short series of stimulation trains, there was a consistent and long-lasting deactivation of forebrain state (33.7±5.0 minutes, n = 4) during which no spontaneous alternations occurred (Figure 11A, D). Thus, stimulation of forebrain-activating brainstem sites implicated in the pacing of cyclical brain state alternations during sleep subsequently disrupts state cycling under urethane anaesthesia. This effect was unlikely to have been mediated by stimulation-induced damage to brain stem sites responsible for forebrain activation since this disruption was only temporary. These effects were also unlikely to have been mediated by prolonged activation of forebrain regions per se since prolonged stimulation of posterior hypothalamic sites, which also potently activated forebrain EEG in a similar way, did not disturb subsequent state alternations (Figure S2).

## Discussion

Our results show that alternations of forebrain state under urethane anaesthesia bear a strong resemblance in their EEG components, evolution, time course, physiological correlates and dependence upon ascending (cholinergic) activating systems to those observed during natural sleep. At a minimum, this implies that urethane induces a pharmacological condition that is similar to natural sleep and constitutes a good model system for the study of brain alternations during unconsciousness.

### Anaesthesia and sleep

The idea that anaesthetics promote unconsciousness by exploiting the brain mechanisms involved in natural sleep, although popular metaphorically [8], has only recently received experimental support. A number of different anaesthetics have been shown to elicit their hypnotic and sedative effects through targeted pharmacological actions upon brain areas important for the elicitation of sleep [38]–[41]. Furthermore, the functional physiological overlap of anaesthetic action and sleep has been demonstrated in experiments that showed that animals not only avoid accruing sleep debt, but are able to recover from sleep deprivation while anaesthetized [42], [43], and that sleep deprived animals are more sensitive to anaesthetic agents [44]. However, it has been normally accepted that all general anaesthetics produce a unitary brain state of unconsciousness similar to nREM (slow-wave) sleep [9]. It is certainly the case in our laboratory that other common veterinary anaesthetics at a variety of surgical anaesthetic dosages do not produce the alternations apparent during urethane anaesthesia [45], [46]. Our present results demonstrate that urethane is unique in its anaesthetic action by producing a form of unconsciousness within which the expression and alternations of different sleep-like states are spontaneously exhibited. In this respect, the pharmacological action of urethane appears similar to the physiological *maintenance* of sleep rather than producing a pharmacological *induction* of a unitary sleep-like slow wave state that may constitute the action of other general anaesthetics.

### Sleep-like cycling under urethane

While urethane has been previously used as a model for the individual brain states of either nREM (including both light and slow-wave stages) or REM sleep [24], [47]–[51], and while both evoked and spontaneous state fluctuations have also been observed to occur with urethane anaesthesia [12]–[14], [34] ours is the first study to demonstrate rhythmic spontaneous state alternations and to directly evaluate and test their resemblance to those of natural sleep. One enabling feature of our studies was the long recording periods used that allowed us to describe the systematic, stereotyped and cyclic repetition of the transitions between these states.

Similar to sleep, the transition between activated and deactivated patterns tended to be a slow process, while the opposite transition tended to be more abrupt. As well, the individual EEG characteristics of the evolution of this transition mimicked components of REM/nREM transitions through lighter and deeper slow wave stages in natural sleep. Furthermore, the period of cyclic state alternations was highly similar to the period of the REM/nREM cycle in natural sleeping rats as previously reported [17] and also as directly assessed in our same animals prior to urethane treatment.

Brain state alternations under urethane were also correlated to other physiological fluctuations that showed further similarities to REM/nREM cycles during natural sleep. Transitions into activated EEG patterns were correlated with significant decreases in EMG tone. This finding is directly comparable to the paralysis that characterizes the REM state during sleep. Interestingly, similar decreases in EMG tone with changes from deactivated to activated states under urethane have been previously reported by other groups [22], [50] and can be directly observed in traces in which this difference was not systematically characterized (see Figure 2 in [52]). As well, like in REM, the activated state under urethane was correlated with an increase in both heart and respiration rates.

In contrast to the interpretation of previous researchers [12]–[14] we found no support for the idea that the alternations present under urethane reflect fluctuations in anaesthetic level. Given that the plane of anaesthesia evoked by urethane is correlated to its blood concentration and that prior studies of the metabolism of urethane have demonstrated a slow and consistent rate of metabolic excretion in rodent blood samples (without any evidence of systematic fluctuations) [15] it is difficult to understand how variations in anaesthetic level might occur in this situation. Indeed, in our experiments, supplemental doses of urethane did not abolish state alternations. Interestingly, and perhaps similar to the effects of sleep deprivation, these manipulations did increase the amount of time per cycle spent in the deactivated state. Furthermore, the net decrease in EMG tone observed in the majority of our EMG recordings (and the lack of change in the remainder) across deactivated to activated transitions is certainly inconsistent with a decrease in anaesthetic level during the activated state. Lastly, and more directly, the withdrawal threshold to painful stimuli was unchanged across both deactivated and activated states.

Related to the above, we also determined that the activated state during urethane was pharmacologically different from that observed during the aroused waking state. It is well known that activated patterns in the forebrain during awake behaviour are dependent upon a host of ascending activating systems including both cholinergic and monoaminergic components [29], [30], [53]. Monoaminergic depletions using reserpine, whether conducted prior or during anaesthesia, were without effect upon the activated state, and indeed, upon the presence of state alternations. Consistent with these findings, the activated state (and moreover state alternations) during urethane anaesthesia was completely abolished by muscarinic receptor antagonism – similar to effects reported for the naturally sleeping animal.

Despite the similarities reported here, there were some obvious differences between urethane anaesthesia and sleep. Firstly, (and as previously acknowledged [9]), sleep and its states are homeostatically regulated, and therefore internally driven. In contrast, the anaesthetized condition was dependent upon circulating levels of exogenously derived urethane. Moreover, anaesthesia (by definition) is a condition from which an organism cannot be “awakened” until the circulating anaesthetic agents have been metabolized or otherwise eliminated. Lastly, the hallmark sign of REM sleep, rapid-eye movements, were absent in the transitions from deactivated to activated patterns of brain state under urethane anaesthesia. This may seem to negate the use of the term “REM-like” to describe the activated state under urethane since rapid eye movements were not observed. However, REM sleep is considered to be made up of two different elements referred to as tonic and phasic [54]. Tonic events are considered to be those that are consistently present during active sleep such as activation of the EEG, muscular atonia, thermoregulatory cessation, etc. Phasic events are those which are only transiently present and include large amplitude pontine derived field potentials (ponto-geniculo-occipital – PGO – waves), rapid eye movements and muscular twitches. Despite this difference, tonic episodes without rapid eye movements are still referred to as components of REM sleep. Perhaps more interestingly in this regard, during urethane anaesthesia we did not observe any phasic events (although the presence of PGO waves was not directly assessed). Indeed, since the brainstem mechanisms responsible for phasic and tonic elements of REM have been shown to be independent [55] it may well be that phasic but not tonic REM events are selectively suppressed by urethane.

Regardless, these differences (along with other dissimilarities such as the eye open posture), suggest - not surprisingly - that the complete spectrum of physiological changes present during natural sleep cycles is not completely mimicked by urethane anaesthesia. However, both the similarities and differences between the urethane and sleeping conditions provide an intriguing means to elaborate the central and peripheral mechanisms involved in the constellation of the physiological correlates of state alternations during unconsciousness themselves.

### Sleep-like brain mechanisms of state alternations under urethane

Although the mechanisms by which state alternations took place under urethane anaesthesia were not explicitly examined in the present study, we found that, like in sleep, they depended on intact cholinergic mechanisms. Perhaps more interestingly, we were able to robustly interfere with ongoing state alternations with relatively moderate trains of electrical stimulation of sites in the brainstem. Although forebrain activation was a prominent effect during stimulation trains, the effect subsequent to a series of trains was a pronounced and lasting deactivation of forebrain regions. This effect was transient since state alternations spontaneously returned after a variable period. This suggests that, like in sleep, the brainstem regions that were stimulated form part of an extended activity-dependent circuit that balances other opposing brainstem elements through mutual, perhaps inhibitory, interactions [27], [28], [35]. It also implies that these interactions, which result in the pacing of the alternations themselves, are subject to perturbation by exogenous stimulation. It is notable in this regard, that even prolonged and strong stimulation trains applied to more rostral brain sites such as the posterior hypothalamus (that forms part of the ascending activating system but is not thought to be involved in pacing state alternations during sleep *per se*) caused no such temporary depression of the ongoing alternations. These findings define an interesting avenue for future study of the brainstem mechanisms involved in state transitions under urethane.

### Pharmacological action and brain targets of urethane

Our findings also underscore the importance of pharmacological approaches designed to pinpoint the mechanisms by which urethane promotes its anaesthetic action [56]–[58]. Knowledge of the brain areas targeted by this drug and its effects on cellular and synaptic physiology would contribute not only to an understanding of the regulation of consciousness and of anaesthetic action, but in the context of our present results would also be relevant for the study and treatment of sleep disorders. One fruitful line of study would be to elucidate the possibility that urethane may have relatively less effect on central cholinergic mechanisms relative to other neuromodulatory systems [30]. Certainly, our results imply that urethane targets brain regions important for the maintenance of consciousness yet does not depress the brainstem and forebrain cholinergic mechanisms giving rise to sleep-like EEG states and their alternations.

### Conclusion

Based upon these data, we consider that urethane anaesthesia constitutes a viable and realistic model system for the alternations of brain state present during natural sleep. This model will undoubtedly aid mechanistic investigations and lead to insights concerning the functional relevance of fluctuations of brain state during unconsciousness. Furthermore, it will potentially facilitate the development of novel sleep agents designed to induce and maintain behavioural unconsciousness without disrupting the brain's natural tendency to fluctuate between different states. The advantage of studying these dynamic events in an anaesthetized preparation, which allows for unfettered control and access, will clearly benefit the field of sleep research and, indeed, neuroscience in general.

## Materials and Methods

Data were obtained from 94 male Sprague-Dawley rats weighing between 171.0 and 410.2 g (262±5.1). All methods used conformed to the guidelines established by the Canadian Council on Animal Care, the Society for Neuroscience, and were approved by the Biosciences Animal Policy and Welfare Committee of the University of Alberta.

### Acute (urethane-anaesthetized) preparation

#### Anaesthesia and surgery

Anaesthesia was initially induced with gaseous isoflurane mixed with medical oxygen at a minimum alveolar concentration (MAC) of 4.0 in an enclosed anaesthetic chamber. Following loss of righting reflexes, they were maintained on isoflurane (2.0 to 2.5 MAC) via a nose cone and implanted with a jugular catheter. Isoflurane was discontinued and general anaesthesia was achieved using slow intravenous (i.v.) administration of urethane (0.8 g/ml; final dosage 1.75±0.04 g/kg). Body temperature was maintained at 37°C using a servo driven system connected to a heating pad and rectal probe (TR-100, Fine Sciences Tools; Vancouver, BC, Canada) for the remainder of the surgical and recording procedures. Level of anaesthesia was assessed throughout the experiment by monitoring reflex withdrawal to a hindpaw pinch.

#### Stereotaxic procedures

Stereotaxic placement of indifferent (reference), recording, and stimulating electrodes in addition to infusion cannulae was conducted using bregma as a coordinate landmark. Recording electrodes were constructed from Teflon-coated stainless steel wire (bare diameter 125 µm: A-M Systems Inc.). These electrodes were aimed at the frontal (AP: +0.3; ML: ±1.0), and posterior (AP: −4.5, ML: ±1.5 mm) neocortices; either in superficial or deep layers (DV: −0.1 to −0.25 or DV: −1.0 to −1.5 mm, respectively). As well, another target was the hippocampal fissure of the dorsal HPC (AP: −3.3, ML: ±2.0, DV: −2.8 to −3.3 mm). Hippocampal signals were recorded since the frequency and power of the theta oscillation is a sensitive index of the degree of forebrain activation [59]. In some experiments additional bipolar electrodes staggered vertically by 1.5–2.00 mm were implanted in frontal cortex (AP: +2.6 mm, ML: ±0.5 mm, DV(deep pole): −1.5–2.0 mm) [47], [60], [61]. Indifferent electrodes (used when not referencing signals to ground) were constructed from either an electrically connected pair of thick Teflon insulated wires (200 µm: A-M Systems Inc. Carlsborg, WA.) staggered by 1.5 mm or a single wire of the same type scraped bare to a distance of at least 1.5 mm and were implanted in the frontal hemisphere (AP: +1.0 mm, ML: ±1.5 mm, DV: −1.5 mm). Indifferent electrodes were verified to be electrically neutral by comparison to ground [10]. Stimulation electrodes were constructed of twisted bipolar Teflon-coated stainless steel wires and were aimed at either the posterior hypothalamus (AP: −3.3 mm, ML: ±0.1 mm, DV: −8.5 mm) or the pedunculo pontine tegmental nucleus (AP:−8.0 mm, ML: ±1.8 mm, DV: −7.8 mm). Cannulae were constructed from blunt ended 30-gauge stainless steel hypodermic needles and were aimed at the basal forebrain region (AP:−0.5 mm, ML: ±2.5 mm, DV: −8.0 mm). In some animals, needle electrodes were implanted bilaterally in the subcutaneous layer of the chest in order to monitor cardiac (EKG) activity. In these same animals, a thermocouple wire (30 gauge Type K; Thermo Electric Co., Inc.; Brampton, ON, Canada) was placed just inside the nasal passage to monitor respiration rate [62]. In other animals electrooculographic (EOG) records were made with teflon-coated silver wire electrodes, implanted in the external canthus muscle as described previously [63]. Following implantation, all electrodes and connecting wires (except for EOG, EKG, and respiration leads) were fixed to the skull using jeweler's screws and dental acrylic.

#### Recording procedures

During recordings, the stereotaxic apparatus was connected to ground. Field potential and EOG recordings were amplified at a gain of 1000 and filtered between 0.1 to 500 Hz using a differential AC amplifier (Model 1700, A-M Systems Inc.). All field signals were referenced to the implanted indifferent electrode or to ground. Comparison of the two configurations ensured that no referencing artefacts ensued [10]. EOG signals were referenced to each other. EKG leads were referenced to each other, amplified at a gain of 1000 and bandpass filtered between 10 to 500 Hz. Thermocouple signals were amplified at a gain of 10000 and filtered between 0.1 and 500 Hz. This method yielded a continuous representation of the respiratory cycle due to the temperature difference between inspired and expired air [62]. All recorded signals were digitized on-line (sampling frequency 1 kHz) with a Digidata 1322A A-D board connected to a Pentium PC running the AxoScope acquisition program (Axon Instruments; Union City, CA).

#### Manipulations

Systemic pharmacological manipulations using atropine sulphate (50 mg/kg), m-oxotremorine (11.17±3.96 mg/kg) and physostigmine (2.39±0.9 mg/kg) were injected either intraperitoneally (i.p.) or i.v. via the jugular or femoral vein. Atropine sulphate and m-oxotremorine are muscarinic antagonists and agonists, respectively while physostigmine is an anticholinesterase (and therefore a cholinergic agonist). Pre-treatment using reserpine (5 mg/kg) involved i.p. injections of the drug 14–18 hours prior to the beginning of the experiment. Reserpine is an antagonist of monoaminergic neurotransmission. Confirmation of its pharmacological effect was established by gross behavioural observations subsequent to the administration in the home cage when the animal displayed behavioural depression characterized by sedation, ptosis, and akinesia [31]–[33]. Site specific inactivation of the basal forebrain was conducted by slowly infusing (1 µl/min) lidocaine (a local anaesthetic) via a microinfusion pump (Model KDS100, KD Scientific Inc. Holliston, Massachusetts) through PE-50 tubing (Fisher Scientific, Ottawa, Ontario) attached to the implanted cannula. Stimulation of the posterior hypothalamus (PH) or pedunculo pontine tegmental nucleus (PPT) was conducted with biphasic square-wave pulses, 0.1 ms in duration at a frequency of 100 Hz for a duration of 5 to 15 seconds (Model 2100, A-M Systems Inc. Carlsborg, Washington). Current amplitudes used ranged from 50 µA to 900 µA. In all experiments, adequate baseline recordings (consisting of at least four complete alternations) were taken prior to manipulations.

Variation in depth of anaesthesia across states was quantified using a nociceptive infrared source producing a heating beam placed beneath the tail or hind paw (Model 7371 Plantar Test, Ugo Basile; Biological Research Apparatus, Comerio, Italy). This device automatically detected withdrawal latency to the nearest 0.1 s. Testing using this apparatus was conducted across alternations in order to acquire withdrawal latencies in both activated and deactivated states.

### Chronic (freely-behaving) preparation

#### Anaesthesia and surgery

In order to implant electrodes for later chronic recordings under natural sleeping conditions animals were anaesthetized with an i.p. injection of a ketamine/xylazine cocktail (90 and 10 mg/kg, respectively). Rats were also administered a subcutaneous dose of atropine methyl nitrate (0.05 mg/kg) to prevent respiratory complications. During anaesthesia, body temperature was maintained at 37°C and level of anaesthesia was assessed as described previously. Supplements of the ketamine/xylazine cocktail (10% of original dose) were administered as necessary to maintain the animal's level of anaesthesia.

#### Stereotaxic procedures

Using antiseptic techniques, animals were implanted stereotaxically (as described above) with unilateral frontal neocortical and bilateral hippocampal electrodes. A wire soldered to a skull screw placed over the cerebellum or an uninsulated electrode implanted vertically in the frontal nCTX served as a signal reference. All intracerebral electrodes were manufactured by Plastics One (Roanoke, VA). Bipolar electromyographic (EMG) electrodes (constructed from the same wire used for field recordings) were implanted in the neck musculature as described elsewhere [64].All wires and connector assemblies were fixed to the skull using jeweller's screws and dental acrylic. Following implantations, the scalp was cleaned and sutured, and the animal was placed in a clean cage.

#### Recovery

Animals were allowed to recover for a minimum of one week prior to any recording. During this time they were handled on a daily basis and habituated to the recording apparatus for at least two hours each day during the light cycle. During the habituation procedure, all leads were connected to suspended wires and animals were allowed to freely behave in the recording chamber that was contained in a Faraday cage and housed in a quiet room. Adequate habituation ensured that animals slept during recording sessions.

#### Recording procedures

Field signals were amplified and digitized as described above. Potentials from bipolar EMG electrodes were referenced to each other, amplified at a gain of 10000 and bandpass filtered between 10 to 500 Hz. Multi-site field recordings were made simultaneously during ongoing (spontaneous) behaviour for a variable time period (1 to 8 hours) daily. Periods of immobility were identified in electrographic recordings by low EMG tone and irregular activity in the HPC. They were classified as sleep only after offline confirmation using temporally correlated video records. Sleep and wake states were distinguished visually using by coding both posture (e.g. head and body down) and eyelid position (e.g. open versus closed). During sleep, REM and nREM episodes could be differentiated by the presence or absence of activated patterns of EEG in the nCTX (low voltage fast activity: LVFA) and HPC (theta), respectively. Further confirmation was obtained with EMG recordings; a decrease in EMG tone was concomitant with nREM to REM alternations.

Following the acquisition of a minimum of 5 complete REM/nREM cycles , rats were prepared for acute (urethane) anaesthetized recordings as described in the previous section. Recordings during anaesthesia were taken using the same connector pins used for naturally sleeping recordings and included EMG.

### Perfusion and histology

Following anaesthetized recording sessions, small lesions were made at the tips of active intracerebral electrodes by passing 0.1 to 1 mA of D.C. current for 5 s using an isolated constant current pulse generator (Model 2100, A-M Systems Inc.). Rats were perfused transcardially, initially with physiological saline, and then with 4% paraformaldehyde in saline. Brains were extracted and stored overnight in 30% sucrose in 4% paraformaldehyde. The tissue was frozen with compressed CO_2_ and sliced at 48 µm with a rotary microtome (Leica 1320 Microtome; Vienna, Austria). Slices were then mounted on gel-coated slides, allowed to dry for a minimum of 24 hours, stained using cresyl violet or thionin, and coverslipped. Microscopic inspection of stained slices was used to verify recording, stimulating and infusion loci [10]. Digital photomicrographs (Canon Powershot S45; Tokyo, Japan) were taken on a Leica DM LB2 (Buffalo, NY) microscope, imported using Canon Remote Capture 2.7 software (Tokyo, Japan) and processed with Corel PhotoPaint (Ottawa, ON, Canada).

### Data processing and analysis

Raw signals were first examined visually using AxoScope (Axon Instruments) and converted to ASCII format for further analysis. Zero-phase distortion digital filtering, single and dual-channel spectral analyses, and auto- and cross-correlations of field, respiration and heart rate activity were conducted offline using Matlab Version 5.1/5.3 (Mathworks; Natick, MA) and visualized using Origin (Microcal Software Inc.; Northampton, MA).

Autopower spectra were computed and plotted for field signals. Static spectra were computed from data segments whose length depended upon the type and stationarity of the signal. For segments of activated patterns with higher frequency components such as theta, segments were at least 30 s. For deactivated patterns with lower frequency components such as LIA and slow oscillations, segments were at least 60 s. Spectra were computed using a series of 6 s long, Hanning-windowed samples with 2 s overlap, across the entire data segment. Spectral values at peak signal frequencies or across a peak frequency bandwidth (comprising a range of 0.3 Hz) were calculated and compared across EEG states. Spectrograms were computed from even longer data segments (several minutes) in which state alternations took place. A sliding windowing procedure was adopted that allowed discrete spectra to be calculated for specific time points across the entire data segment. Windows were 24 s in duration and were moved across the data segment in increments of 6 s. Spectral analysis of these individual samples was identical to the methods described above. Period analysis was performed by creating a sliding spectrogram of acquired data to visualize dynamics of all frequencies across time. The power at 1 Hz was extracted from this data and plotted separately since it showed the highest fluctuation across state changes. This method facilitated the temporal (period) analysis of state changes.

To identify spindles, we used methods similar to previous investigators [65]. In brief, we decimated data for a sampling frequency of 100 Hz, and applied a band-pass filter of 7–10 Hz to cortical traces. The root mean square (RMS) value was calculated at every time point using a window of 0.2 seconds. Both the mean and standard deviation (SD) of RMS were calculated for individual files. A threshold value (mean+3 SD) was set to identify spindles. Duration of a spindle was acquired by calculating the time difference between when the RMS superseded a value of one SD greater than the mean prior to and after crossing the threshold value. Any spindles that had a duration of less than 0.5 s were discarded. The temporal occurrence of spindles relative to the evolution of the activated-deactivated alternations was assessed by extracting the spectrographic power at 7, 8 or 9 Hz and comparing it to the 1 Hz spectrographic power (described above). The temporal relationship between the two time series power signals was explicitly examined using cross-correlation analysis. The data were summarized across experiments by standardizing the alternation periods as a complete cycle (i.e. from 0 – 360 degrees) and creating an average cross-correlation.

Heart and respiration rates were also quantified using spectrographic measures, with a frequency resolution of 0.05 Hz. Peak frequencies were extracted and plotted across time. Both the peak frequency values and their temporal variations (assessed by differentiation) were compared across states and state alternations.

EMG analysis consisted of peak-to-peak quantification across states. Normalization across animals, for the purpose of pair-wise comparisons, was done by representing the activated or REM state as a percentage of the value for the deactivated or nREM state.

Data designed to assess behavioural level of anaesthesia were organized across time, stimulus presentation, and state. Comparisons of withdrawal latency across states was conducted for adjacent datasets in time and stimulus presentation using two sample t-tests (p<0.05). Care was taken to ensure that no systematic changes in the sensitivity of the behavioural response as a function of repeated stimulus presentation occurred by performing linear regression within and between datasets. No t-test comparisons were made for datasets showing significant (p<0.05) linear relationships with time or stimulus number.

Summary reports of data across experiments or conditions in the Results section are reported as arithmetic means together with the standard error of the mean (SEM) unless otherwise noted. Numerical comparisons across conditions for the same datasets were made using pairwise t-tests (with a significance level of 0.05). As most tests were specified a priori, this was also the case for datasets with more than two conditions. For remaining situations, data were analyzed with one-way ANOVA (using the Tukey post-hoc corrected tests) analyses with significance at a level of 0.05.

### Drugs and chemicals

Atropine methyl nitrate, atropine sulphate, reserpine, physostigmine, lidocaine, m-oxotremorine, ethyl carbamate (urethane), and thionin were all purchased from Sigma (St. Louis, MO). All reagents were mixed in double distilled water. Reserpine solutions were made by first dissolving 10 mg in 50 µL glacial acetic acid, then adding double distilled water to make a final solution of 1 ml. Isoflurane and ketamine were purchased from Bimeda-MTC (Animal Health Inc.; Cambridge, ON, Can). Cresyl violet was purchased from Acros Organics (Morris Plains, NJ), paraformaldehyde from Fisher Scientific (Toronto, ON, Can), and xylazine from Bayer Inc. (Toronto, ON, Can).

## Supporting Information

Figure S1 State alternations were dependent upon central cholinergic neurotransmission. A) Ultra long duration cortical and hippocampal EEG traces in addition to spectrographic cortical power at 1 Hz demonstrating the effects of agonism and subsequent antagonism of cholinergic transmission. Following an i.v. injection of physostigmine (3.7 mg/kg) spontaneous alternations between activated and deactivated states were temporarily abolished in favor of the activated state. Following recovery, state alternations were permanently abolished in favor of the deactivated state with a subsequent i.p. injection of atropine sulfate (ATSO4: 50 mg/kg). B) Expansions of EEG traces from neocortical and hippocampal sites show the similarity of activated and deactivated patterns induced by cholinergic agonism and antagonism, respectively. C) Scatter plots demonstrating the duration of effects of physostigmine and atropine as a function of dosage. The effect of atropine never washed out even following lengthy subsequent recordings.
(1.53 MB TIF)

Figure S2 Even intense stimulation trains applied to the posterior hypothalamus did not abolish subsequent alternations of forebrain state. A) Continuous EEG traces and the spectrographic cortical power at 1 Hz demonstrating the effects of stimulation of the posterior hypothalamic (PH) region. Following a stimulation train that was applied through an entire cycle, spontaneous state alternations returned to normal. B) Expanded EEG traces from neocortical and hippocampal sites demonstrate that activated patterns were elicited via stimulation of the PH region. C) Scatterplot and linear fit of frequency as a function of the stimulation intensity in the PH showing a significant (p<0.01) relationship between stimulation intensity and the peak frequency of theta activity recorded in the hippocampus. The frequency was normalized across experiments to the maximal frequency of theta elicited in each. D) Summary of histological findings for the sites of stimulation for every experiment. Abbreviations: DM: dorsal medial hypothalamic nucleus, ec: external capsule, f: fornix, fi: fimbria, H: habenular nucleus ic: internal capsule, mt: mammilothalamic tract, PH: posterior hypothalamus, PVP: paraventricular thalamic nucleus, St: stria terminalis.
(2.45 MB TIF)
