# Supplementary figures and images for: Cyclic and Sleep-Like Spontaneous Alternations of Brain State Under Urethane Anaesthesia
Source: PLoS One. 2008 Apr 16;3(4):e2004. doi: 10.1371/journal.pone.0002004 (PMC2289875; doi:10.1371/journal.pone.0002004)

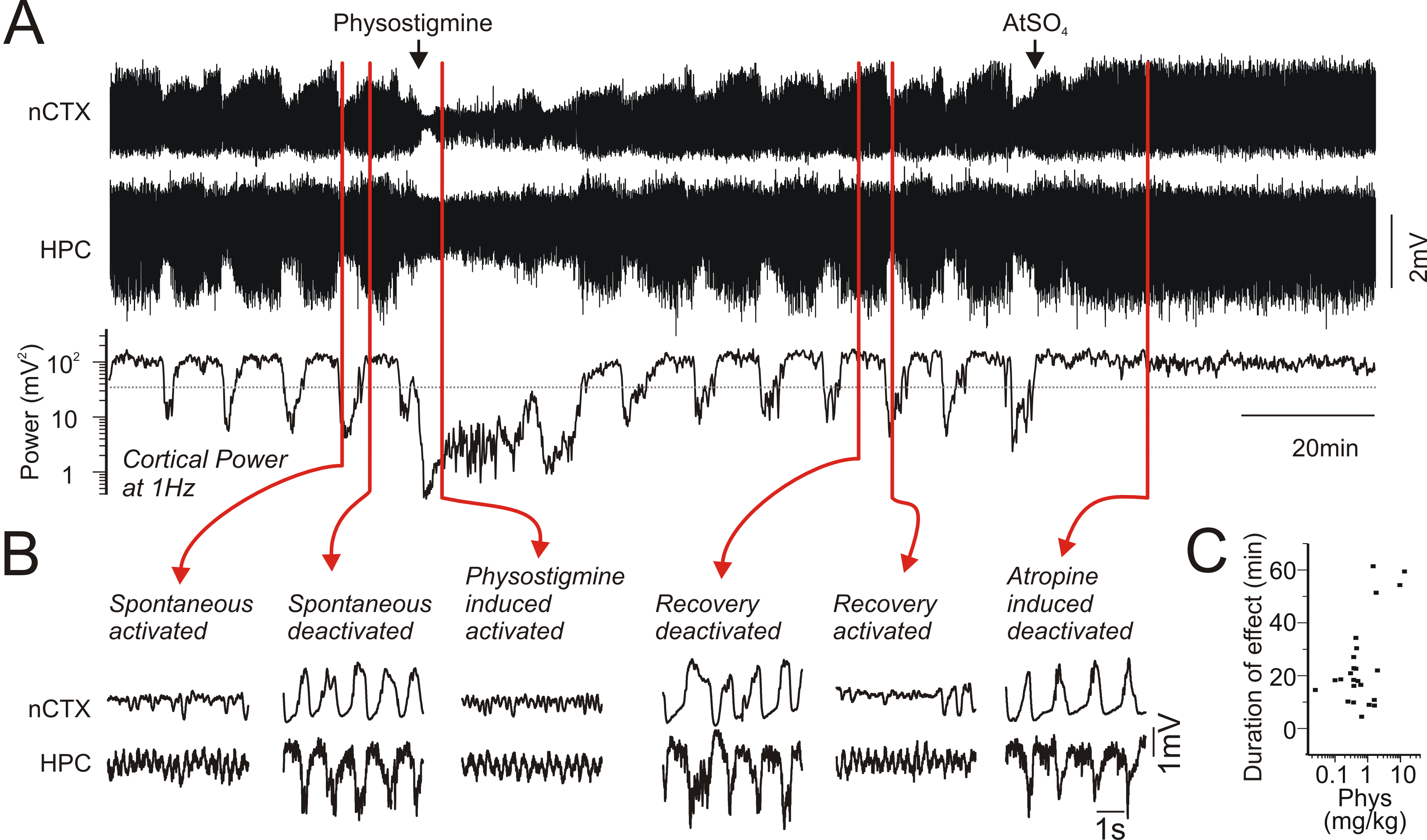

Supplement: Figure S1 — State alternations were dependent upon central cholinergic neurotransmission. A) Ultra long duration cortical and hippocampal EEG traces in addition to spectrographic cortical power at 1 Hz demonstrating the effects of agonism and subsequent antagonism of cholinergic transmission. Following an i.v. injection of physostigmine (3.7 mg/kg) spontaneous alternations between activated and deactivated states were temporarily abolished in favor of the activated state. Following recovery, state alternations were permanently abolished in favor of the deactivated state with a subsequent i.p. injection of atropine sulfate (ATSO4: 50 mg/kg). B) Expansions of EEG traces from neocortical and hippocampal sites show the similarity of activated and deactivated patterns induced by cholinergic agonism and antagonism, respectively. C) Scatter plots demonstrating the duration of effects of physostigmine and atropine as a function of dosage. The effect of atropine never washed out even following lengthy subsequent recordings. (1.53 MB TIF) [file pone.0002004.s001.tif]

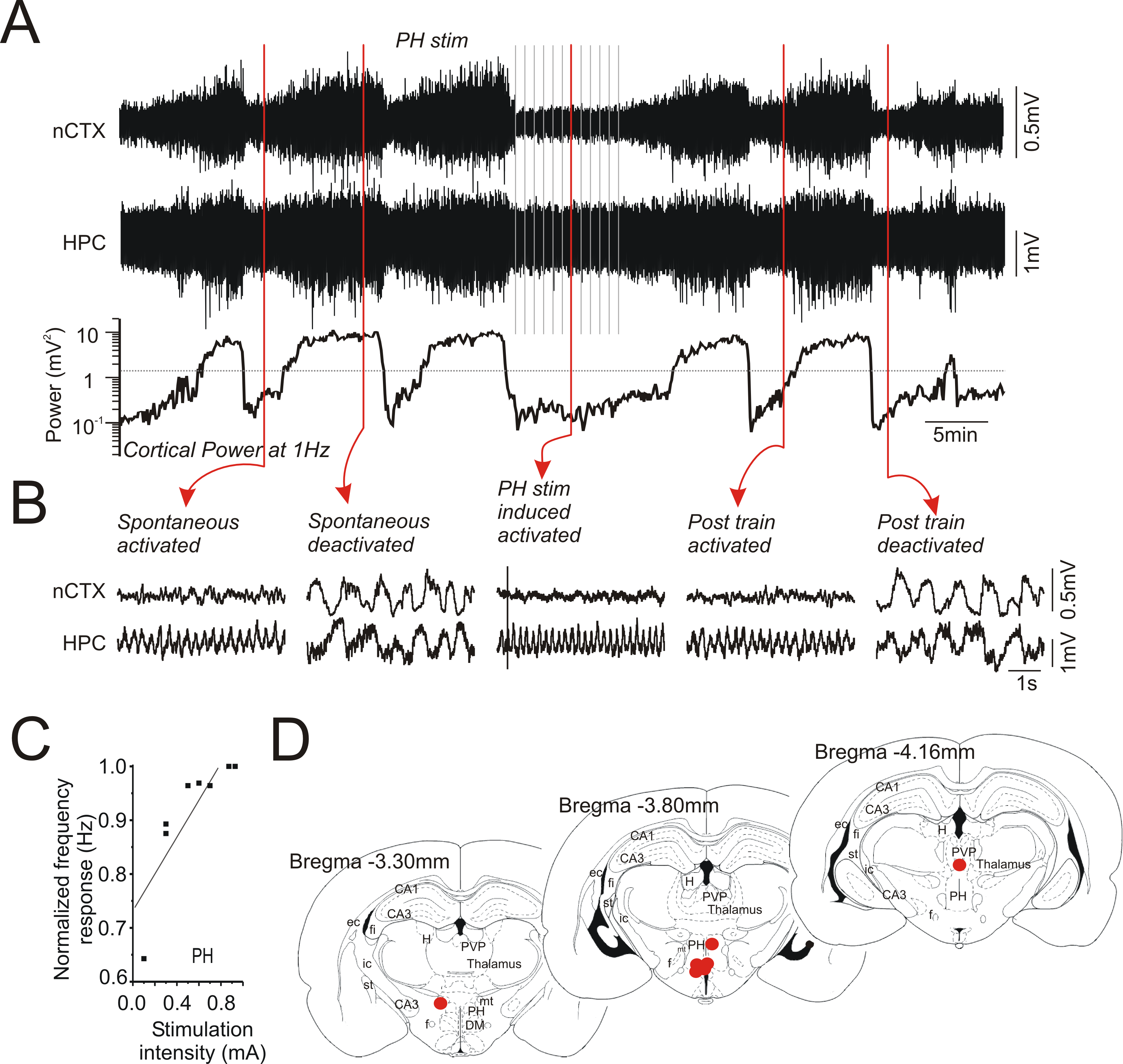

Supplement: Figure S2 — Even intense stimulation trains applied to the posterior hypothalamus did not abolish subsequent alternations of forebrain state. A) Continuous EEG traces and the spectrographic cortical power at 1 Hz demonstrating the effects of stimulation of the posterior hypothalamic (PH) region. Following a stimulation train that was applied through an entire cycle, spontaneous state alternations returned to normal. B) Expanded EEG traces from neocortical and hippocampal sites demonstrate that activated patterns were elicited via stimulation of the PH region. C) Scatterplot and linear fit of frequency as a function of the stimulation intensity in the PH showing a significant (p<0.01) relationship between stimulation intensity and the peak frequency of theta activity recorded in the hippocampus. The frequency was normalized across experiments to the maximal frequency of theta elicited in each. D) Summary of histological findings for the sites of stimulation for every experiment. Abbreviations: DM: dorsal medial hypothalamic nucleus, ec: external capsule, f: fornix, fi: fimbria, H: habenular nucleus ic: internal capsule, mt: mammilothalamic tract, PH: posterior hypothalamus, PVP: paraventricular thalamic nucleus, St: stria terminalis. (2.45 MB TIF) [file pone.0002004.s002.tif]
